# Resource-dependent foraging behaviour of grazers enhances effects of nutrient enrichment on algal biomass

**DOI:** 10.1007/s00442-022-05308-3

**Published:** 2023-01-06

**Authors:** Alessandra Iannino, Patrick Fink, Alexander Tim Ludwig Vosshage, Markus Weitere

**Affiliations:** 1 Department of River Ecology, Helmholtz Centre for Environmental Research - UFZ, Brückstraße 3a, 39114 Magdeburg, Germany; 2 Workgroup Aquatic Chemical Ecology, University of Cologne, Zülpicher Straße 47B, 50674 Cologne, Germany; 3 Department of Aquatic Ecosystem Analysis and Management, Helmholtz Centre for Environmental Research - UFZ, Brückstraße 3a, 39114 Magdeburg, Germany

**Keywords:** Eutrophication, Foraging areas, Food quality, Movement ecology, Periphyton

## Abstract

Both the quantity and nutritional quality of food resources can strongly influence the foraging movements of herbivores, which in turn determine the strength of top-down control on primary producer biomass. Nutrient enrichment can alter the biomass and nutritional quality of primary producers, but the consequences for the foraging of herbivores and hence for top-down control are still poorly understood. In this study, we combined a two-factorial experiment (two nutrient levels × grazing by the freshwater gastropod *Ancylus fluviatilis*) with video analyses tracking grazers’ movements to investigate nutrient enrichment effects on spatial ranges of grazing activity and algal biomass removal. Natural stream biofilms were grown in phosphorus-enriched (P+) and phosphorus-poor flumes (P−) for two weeks before *A. fluviatilis* were added to the flumes and allowed to graze on biofilm for an additional 2 weeks. Total periphyton biomass was enhanced by P+ and reduced by grazer presence. However, the total grazer effect depended on the nutrient level: at the end of the experiment, on average 95% of algal cover were removed by grazing in the P− flumes versus 26% in the P+ flumes. Fast movements of *A. fluviatilis* were detected significantly more often in the P− treatment, whereas grazers were detected resting more often in the P+ treatment. Our results demonstrate that nutrient enrichment can increase primary producer biomass both directly and indirectly by limiting the foraging ranges of herbivores. The resulting feedback loop between reduced grazing activity and increased plant biomass might in turn exacerbate eutrophication effects on habitat structure.

## Introduction

Grazers typically move within landscapes to search for food resources, and their foraging movements can considerably influence the spatial structure of an environment (Adler et al. 2001; Augustine and Frank 2001). Several factors, both biotic (e.g. predation) and abiotic (e.g. physical constraints) can affect the movement behaviour of grazers, thereby creating feedbacks between grazing activity and resource distribution within a landscape (Bailey et al. 1996; Bailey and Provenza 2008). According to optimal foraging models, grazers should adopt feeding behaviours that maximise net energy and nutrient intake (Pyke 1984; Wilson et al. 2012). Therefore, food density and nutritional value can strongly affect herbivore foraging movements and residence times in feeding stations, as food quantity and quality are both limiting factors for herbivore fitness (Fink and von Elert 2006).

The abundance and nutritional quality of primary producers is strongly influenced by nutrient supply (bottom-up control), which has been increasing in the past decades as a result of human activities, leading to eutrophication in both aquatic (Wurtsbaugh et al. 2019) and terrestrial ecosystems (Clark et al. 2017). Elevated inputs of phosphorus (P) and nitrogen (N) commonly increase the productivity of primary producers and their nutrient content relative to carbon (C), as well as alter the species composition of algal and plant communities (Smith et al. 1999; Elser et al. 2007; Sardans et al. 2012). Grazing activity of herbivores (top-down control) can counteract the excessive growth of primary producers caused by nutrient enrichment (Hillebrand 2002; Borgström et al. 2017; Anderson et al. 2018). The foraging behaviour of grazers depends on both resource quantity and quality, and different groups of aquatic invertebrates have evolved behavioural strategies to cope with food and nutrient limitation (Cruz-Rivera and Hay 2000; Moelzner and Fink 2014). For example, high nutrient supply has been shown to reduce the strength of top-down control of algal biomass by decreasing herbivore consumption rates of nutrient-enriched food (Iannino et al. 2019). Yet, the effects of nutrient enrichment on the foraging movements of herbivores, and their consequences for the spatial structure of an environment are still poorly understood.

Interest in movement ecology has been recently increasing, thanks to advances in tracking technology (Nathan et al. 2008; Wilmers et al. 2015). Studies on long-term foraging movements in terrestrial ecosystems have shown that herbivores spend longer times in more profitable food patches, both in terms of food quality and quantity, in accordance with optimal foraging models (Searle et al. 2005; Courant and Fortin 2012). Moreover, foraging movements of terrestrial herbivores are slower when resources are more abundant (Owens-Smith et al. 2010), whereas dispersal is often prompted by low food availability (Bowler and Benton 2005). In aquatic ecosystems, benthic grazers that feed on spatially structured attached algae (periphyton) are comparable to terrestrial herbivores in their foraging movements, in contrast to plankton feeders that consume suspended particles. However, unlike terrestrial plants, periphytic algae allow for experimental manipulation over multiple generations in a relatively short time. Periphyton is thus an ideal model to study feedback effects between grazing activity and the spatial distribution and abundance of primary producers.

To investigate how nutrient enrichment affects grazer movements and hence the spatial extent of algal biomass removal by grazing, we tracked the foraging movements of a stream benthic herbivore, the pulmonate gastropod *Ancylus fluviatilis* (river limpet), in environments of different nutrient status. The following hypotheses were tested: (1) addition of phosphorus as limiting nutrient would lead to both higher algal biomass and nutritional quality, measured as phosphorus content relative to carbon (C:P stoichiometry); (2) as a result of the increased periphyton quantity and quality, grazers would move more slowly and less frequently to search for food in phosphorus-rich (P+) than phosphorus-poor (P−) flumes; and (3) grazed areas would be smaller at high phosphorus relative to low phosphorus supply.

## Materials and methods

### Experimental set-up and procedure

The experiment was performed in the MOBICOS mesocosm facility, a container-based laboratory equipped with flumes fed by a river bypass (Fink et al. 2020) located by the river Holtemme in Wernigerode, central Germany (51°49′00.7″ N, 10°43′29.26″ E) at a relatively pristine site directly downstream of the Harz National Park. At this site, the river still has full canopy cover from riparian broadleaf vegetation and moderate dissolved organic carbon (mostly refractory DOC) and inorganic nitrogen concentrations (approx. 1.5 mg NO_3_ L^−1^, for details see station no. 3 in Weitere et al. 2021). As a consequence, algal C:N ratios do not respond to changes in nitrogen availability, and phosphorus (< 3 µg L^−1^ ambient soluble reactive phosphorus, SRP) and light availability are the main limiting factor for primary production at this site (Fink et al. 2020; Weitere et al. 2021).

Each experimental unit consisted of a rectangular flume (62 cm long, 14 cm high and 8 cm wide) constantly supplied with unfiltered water from the river Holtemme with a flow rate of 1100 L h^−1^ per flume (approximately representative of baseflow conditions of the Holtemme River, Weitere et al. 2021. The water level in each flume was 7.5 cm. A tray containing 30 white ceramic tiles (2.3 × 2.3 cm, for a total of 158.7 cm^2^), disposed in three rows of 10 tiles each, was placed at the bottom of each flume. The tiles served as standardised substrates for periphyton growth. Vertical nets were placed at both ends of each flume to prevent grazers from leaving the experimental facility. The light (PAR) intensity above the flumes, produced by LED lamps, was 54.33 µmol m^−2^ s^−1^ in a 12:12 h light:dark cycle.

The study consisted of a fully factorial experiment, in which two levels of phosphorus supply (P+, P−) were crossed with grazer presence (G+) and absence (G−), for a total of four treatments: P+G+, P+G−, P−G+, and P−G−. In the P− treatments, the water flowing in the flumes was kept at ambient P concentration, which was below detection limit (see above). In the P+ treatments, a concentration of 60 µg P L^−1^ was achieved in each flume by pumping a constant supply of dissolved KH_2_PO_4_ with a peristaltic pump. This elevated SRP concentration was regularly validated with photometric quick-tests (Hach LCK350). In the G+ treatments, eight similarly sized adult individuals (shell length approx. 5 mm) of the pulmonate freshwater limpet *Ancylus fluviatilis*, collected from the Holtemme River near the study site, were added to each flume. Each of the four treatments was replicated three times, for a total of 12 flumes.

Natural periphyton was pre-cultivated for 2 weeks in the experimental flumes at low and high P supply, corresponding to the P− and P + treatments, from 9 to 22 October 2018 before adding grazers. On 22 October, six periphyton-covered tiles were selected from each flume for initial sampling. Periphyton was scraped off each tile, homogenised in tap water and filtered onto pre-combusted glass fibre filters for dry mass, elemental and pigment analyses (see following sections).

On 23 October, eight *A. fluviatilis* individuals were added to each G+ flume and allowed to graze on periphyton for two weeks. Grazer movements were monitored with cameras mounted above the flumes. On 7 November, all grazers were removed from the flumes. The remaining periphyton was scraped off each tile, homogenised in tap water and filtered onto pre-combusted glass fibre filters for dry mass, elemental and pigment analyses. Grazing rates were quantified for P+ and P− flumes by calculating the difference between the average periphyton dry mass in the grazer-free flumes and the remaining periphyton dry mass in the grazed flumes.

### Grazer movement tracking and grazed areas

The video-monitoring set-up above each flume consisted of a Raspberry Pi 3 computer (www.raspberrypi.org) equipped with a Raspberry Pi camera module. Each camera was mounted on a wooden stand to allow aerial perspective recordings of the respective flume. To prevent distortion of recorded pictures (e.g. by water surface movements or light reflections), a transparent Perspex plate was mounted in each flume touching the water surface.

The movement tracking of grazers was based on object detection by colour. Therefore, each grazer shell was marked with a dot of pink nail polish. Every 15 s, each camera recorded a picture that was subsequently used as frame in the output video for each flume. Objects of pink pixels were detected on each frame with the Python package cv2 (Open CV, Open Source Computer Vision Library, 2015) via RGB colour code matching. The x- and y- coordinates of each object were noted for each picture to mark the position of grazers. Each grazer was identified by selecting the objects with the shortest Euclidean distance from each other in consecutive frames, which is easily accomplished for a slow-moving gastropod such as *A. fluviatilis*. Eventually, the coordinates of all grazers over time were used to calculate the speed of each movement according to the time interval between frames. Grazer movements for each frame interval were subsequently classified as either fast (> 4 cm h^−1^), slow (0.4–4 cm h^−1^) or resting (0–0.4 cm h^−1^). In addition, grazed areas over time were quantified by analysing a frame for each flume every 12 h (at approximately 8:00 AM and 8:00 PM every day of the experiment), selecting grazed and non-grazed areas in the frame and converting pixels into cm^2^ according to the known tile dimensions. Grazed areas were clearly distinguishable from the non-grazed areas due to the significant biofilm loss.

### Periphyton dry mass and C:P analysis

For dry mass analysis, periphyton samples were filtered onto pre-weighed glass fibre filters, which were dried at 60 °C for 24 h and weighed with a microbalance to the nearest µg. Periphyton C content was measured with a Vario EL Cube elemental analyser (Elementar Analysensysteme GmbH, Hanau, Germany). For P analysis, filters were placed in a solution of 9% potassium peroxodisulphate and 0.9% sulphuric acid, and heated at 100 °C for one hour in a DigiPREP Block Digestion System (SCP Science, Quebec, Canada). Periphyton particulate P was subsequently analysed with the molybdate-ascorbic acid method (Greenberg et al. 1985) with a DR5000 UV–Vis spectrophotometer (Hach, Düsseldorf, Germany).

### Pigment extraction and analysis

Pigment analysis was performed to estimate the algal community composition of periphyton based on marker pigment:chlorophyll *a* ratios (Lauridsen et al. 2011; Schlüter et al. 2016). For pigment extraction, filters were transferred in 96% ethanol, left at room temperature for 2 h and stored overnight at − 20 °C. The freezing–thawing cycle was performed twice. Subsequently, the sample tubes were placed in an ultrasound bath for 1 min and centrifuged to remove filter residues.

The samples were analysed via high performance liquid chromatography with a Thermo Scientific UltiMate 3000 HPLC System (Dionex, Thermo Fisher Scientific Corporation, Waltham, MA, USA). Pigments were separated with a reverse phase YMC C30 column. The two solvents used were composed of 45:20:30:5 methanol:acetonitrile:water:ion pair reagent (ammonium acetate + tetrabutylammonium acetate) and 30:50:20 methanol:acetonitrile:ethyl acetate, respectively. The flow gradient was the following: 0–4 min: 80% solvent A, 20% solvent B; 35 to 75 min: 100% solvent B; 77 to 80 min: 80% solvent A, 20% solvent B. The flow rate was 0.2 ml min^−1^ and the column oven was set at 35 °C.

Pigment measurements were used to determine the community composition of periphyton with CHEMTAX (version 1.95, Wright and Mackey, Hobart, Australia) according to Mackey et al. (1996) and Lauridsen et al. (2011). The pigment:chlorophyll *a* ratio matrix for meso-eutrophic environments from Schlüter et al. (2016) was used as input ratio matrix for the P+ treatments, and the ratio matrix for oligotrophic environments was used for the P− treatments. CHEMTAX generated 60 ratio matrices from the input matrix for each treatment. Of these, the six matrices (10%) with the lowest residual root mean square were selected and averaged to create a final ratio matrix for each treatment, which was run repeatedly until the ratios and root mean square were stable. The final results from CHEMTAX gave an estimation of the community composition of each sample, in terms of contribution to the total chlorophyll *a* from each algal group (Mackey et al. 1996; Lauridsen et al. 2011; Schlüter et al. 2016).

### Statistical analyses

Statistical analyses were performed in R (R Core Team, version 3.6.1 2019). All data were checked for normal distribution with a Shapiro–Wilk’s test and for homoscedasticity with a Levene’s test.

Before grazers were added to the flumes, periphyton C:P ratios and dry mass were compared between the P+ and P− treatments with a student’s *t*-test, whereas a Welch’s *t*-test was used to compare the relative abundance of diatoms and chlorophytes (derived from CHEMTAX) between the two treatments, as these data were normally distributed but did not display homogeneity of variance. At the end of the grazing phase, interactive effects of P supply and grazing on periphyton dry mass, C:P ratio, and relative abundance of diatoms and chlorophytes were determined with two-way ANOVAs followed by Tukey’s HSD post hoc tests. In addition, student’s *t*-tests were used to compare final grazed areas, grazer resting times and grazing rates between the P+G+ and P−G+ treatments (i.e. P + and P− treatments in the presence of grazers). Wilcoxon–Mann–Whitney tests were used to compare fast and slow movements of grazers between the P+G+ and P−G+ treatment, as these data were not normally distributed.

## Results

### Periphyton properties

**Fig. 1:**
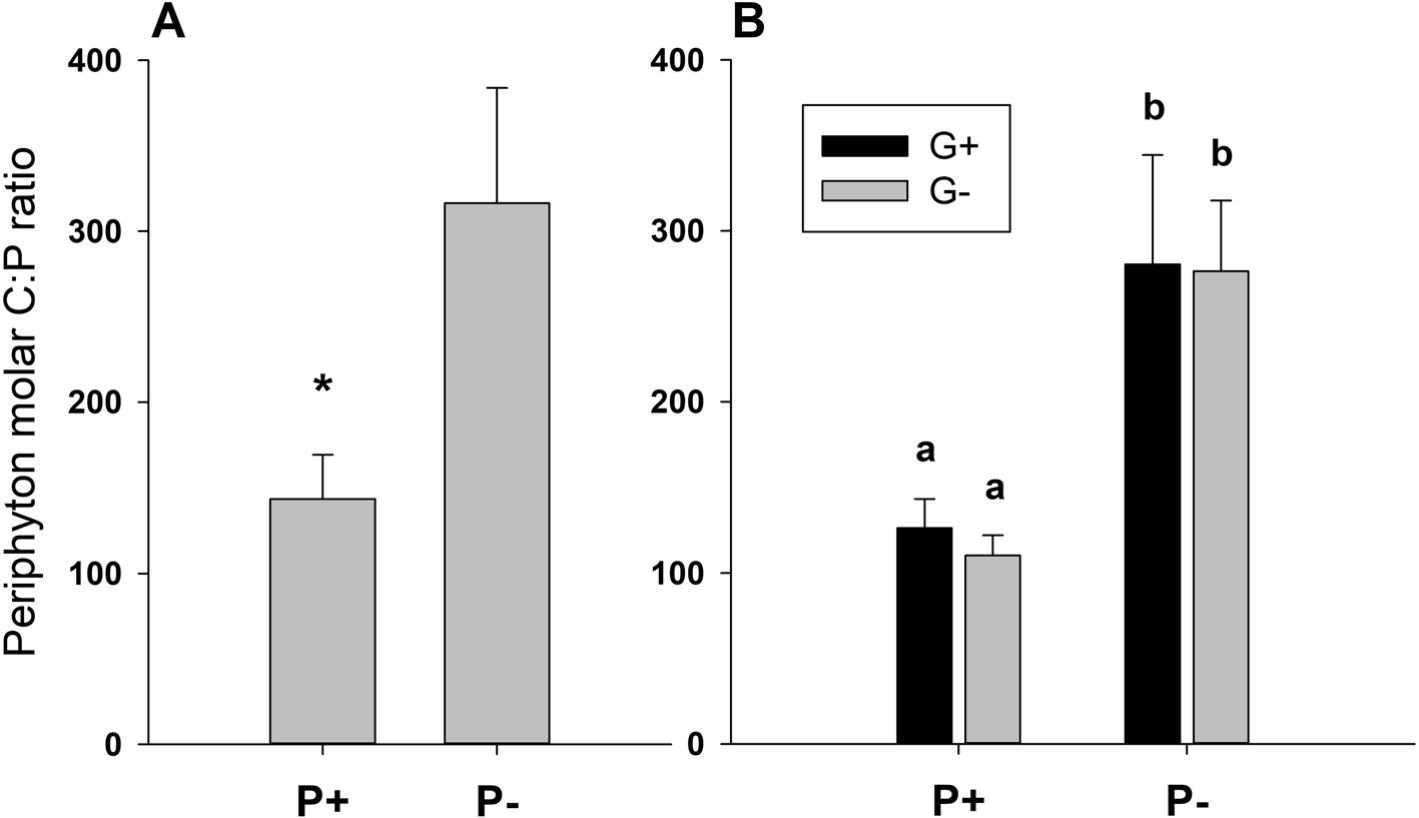
Periphyton molar C:P ratio at test start (**A**) and end (**B**) in the P+ and P− flumes in the presence and absence of grazers (G+ and G−, respectively). Values are mean ± SD of *n* = 6 (**A**) and *n* = 3 (**B**) replicate flumes. P addition significantly decreased C:P ratios both at test start (student’s *t*-test; *t* = − 5.85, df = 10, *p* < 0.001) and at test end (two-way ANOVA; *F*_1, 8_ = 49.46, *p* < 0.001). Significant differences between treatments are indicated by an asterisk in panel **A** and by different letters in panel **B**

**Fig. 2:**
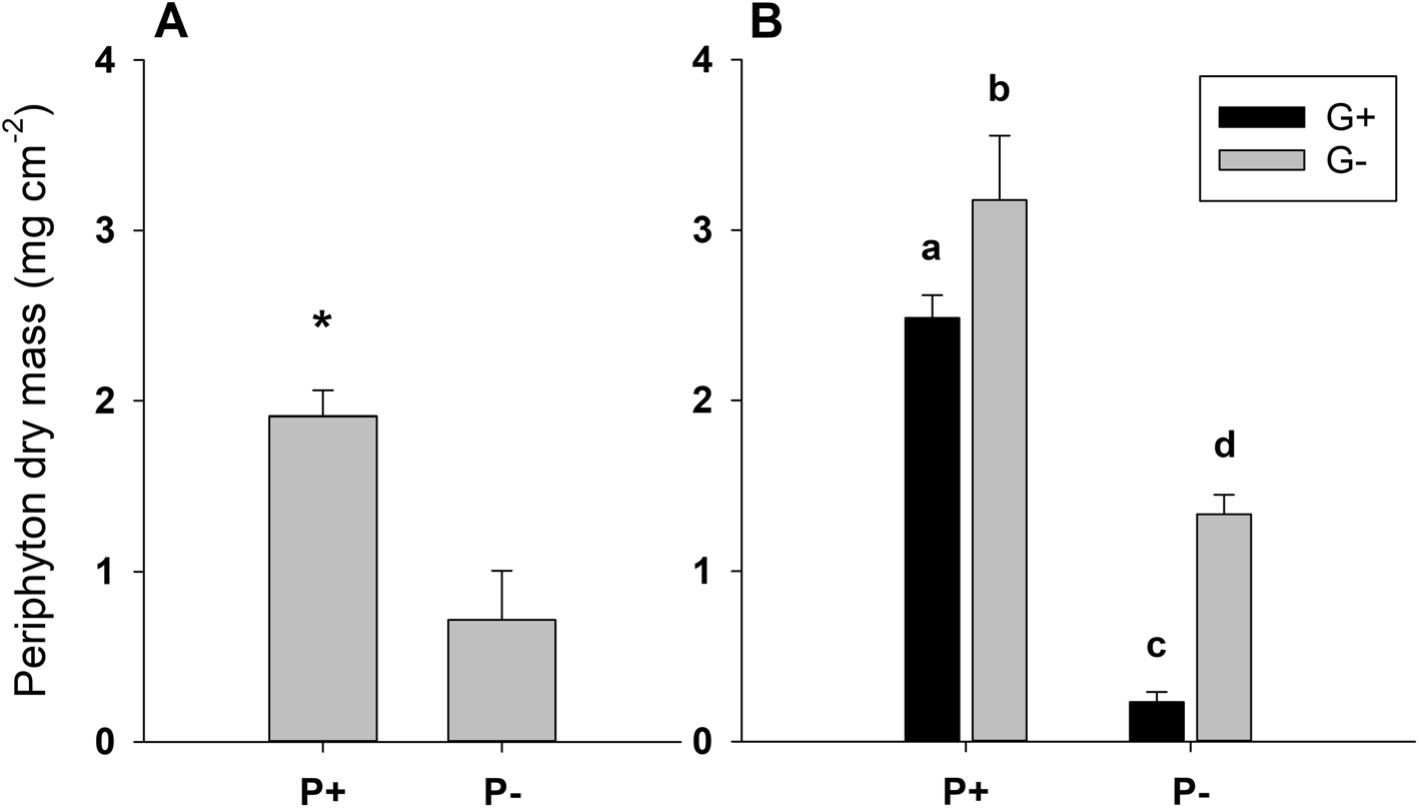
Periphyton dry mass at test start (**A**) and end (**B**) in the P+ and P− flumes in the presence and absence of grazers (G+ and G−, respectively). Values are mean ± SD of *n* = 6 (**A**) and *n* = 3 (**B**) replicate flumes. P addition significantly increased dry mass both at test start (student’s *t*-test; *t* = 8.94, df = 10, *p* < 0.001) and at test end (two-way ANOVA; *F*_1, 8_ = 283.50, *p* < 0.001). Grazing activity significantly reduced periphyton dry mass at test end (two-way ANOVA; *F*_1, 8_ = 54.50, *p* < 0.001). Significant differences between treatments are indicated by an asterisk in panel **A** and by different letters in panel **B**

**Fig. 3:**
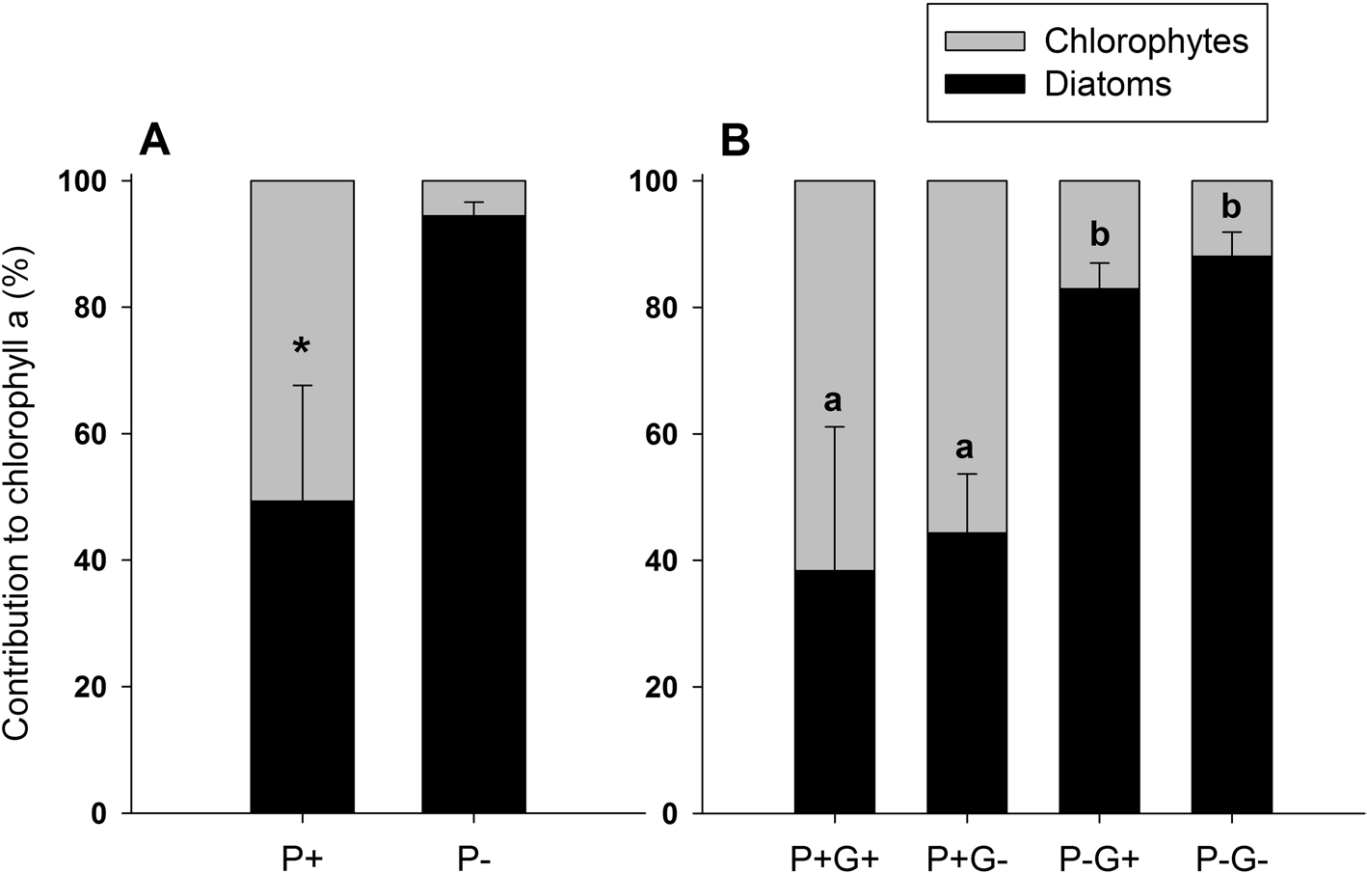
Periphyton relative abundance of diatoms and chlorophytes, measured as percentage of total chlorophyll *a*, at test start (**A**) and end (**B**) in the P+ and P− flumes in the presence and absence of grazers (G+ and G−, respectively). Values are mean ± SD of *n* = 6 (**A**) and *n* = 3 (**B**) replicate flumes. P addition significantly increased the proportion of chlorophytes over diatoms, both at test start (Welch’s *t*-test; *t* = − 5.989, df = 5.14, *p* = 0.002) and at test end (two-way ANOVA; *F*_1, 8_ = 36.75, *p* < 0.001). Significant differences between treatments are indicated by an asterisk in panel **A** and by different letters in panel **B**

At the start of the grazing phase, i.e. after 14 days of grazer-free periphyton growth, phosphorus addition resulted in significantly lower periphyton C:P ratios (student’s *t*-test; *t* = − 5.85, df = 10, *p* < 0.001; Fig. 1A). The average molar C:P ratio of periphyton grown under P+ and P− conditions was 144 ± 26 and 316 ± 68 (mean ± SD), respectively (Fig. 1A). Periphyton dry mass was approximately three times higher in the P+ treatment with a mean of 1.91 ± 0.15 mg cm^−2^, compared to periphyton in the P− treatment with a mean of 0.72 ± 0.29 mg cm^−2^ (student’s *t*-test; *t* = 8.94, df = 10, *p* < 0.001; Fig. 2A). The periphytic algal community was dominated by diatoms and chlorophytes, with P addition significantly increasing the proportion of chlorophytes versus diatoms (Welch’s *t*-test; *t* = − 5.989, df = 5.14, *p* = 0.002; Fig. 3A). Under P− conditions, diatoms contributed to an average of 94 ± 2% of the total periphyton chlorophyll *a*, whereas the P+ periphytic community was on average composed of 49 ± 18% diatoms and 51 ± 18% chlorophytes (Fig. 3A).

At the end of the experiment, i.e. after 14 days of grazing, periphyton C:P ratios were still significantly lower in the P+ treatment, but unaffected by grazing (two-way ANOVA, Table 1, Fig. 1B). Periphyton dry mass was significantly affected by both P addition and grazing (two-way ANOVA; Table 1), but no interaction of the two factors was detected (Fig. 2B). Under P+ conditions, periphyton dry mass increased to an average of 3.18 ± 0.38 mg cm^−2^ in the grazer-free treatment and to 2.48 ± 0.13 mg cm^−2^ in the grazing treatment (Fig. 2B). Under P− conditions, periphyton dry mass increased to an average of 1.33 ± 0.11 mg cm^−2^ in the grazer-free treatment, but decreased to 0.23 ± 0.06 mg cm^−2^ in the grazing treatment, which is lower than the dry mass at the start of the grazing phase. In the P- treatment, grazing was hence responsible for an 83% reduction of periphyton dry mass, while grazing reduced periphyton dry mass by 22% in the P+ treatment compared to the grazer-free control (Fig. 2B). The proportion of diatoms over chlorophytes was still significantly lower in the P+ treatment compared to the P− treatment (Fig. 3B), but it was not affected by grazing (two-way ANOVA, Table 1).

**Table 1:** Results of two-way ANOVAs on the effects of phosphorus and grazing on periphyton C:P ratio, dry mass and diatom relative abundance (as percentage of total chlorophyll *a*)

	Phosphorus		Grazing		Phosphorus × Grazing	
	*F*_*1,8*_	*p*	*F*_*1,8*_	*p*	*F*_*1,8*_	*p*
C:P	49.46	** < 0.001**	0.19	0.68	0.07	0.80
Dry mass	283.50	** < 0.001**	54.50	** < 0.001**	2.83	0.13
Diatom %	36.75	** < 0.001**	0.59	0.47	0.004	0.95

Significant effects (*p* < 0.05) are highlighted in bold

### Spatial impact of grazing on periphyton cover

**Fig. 4:**
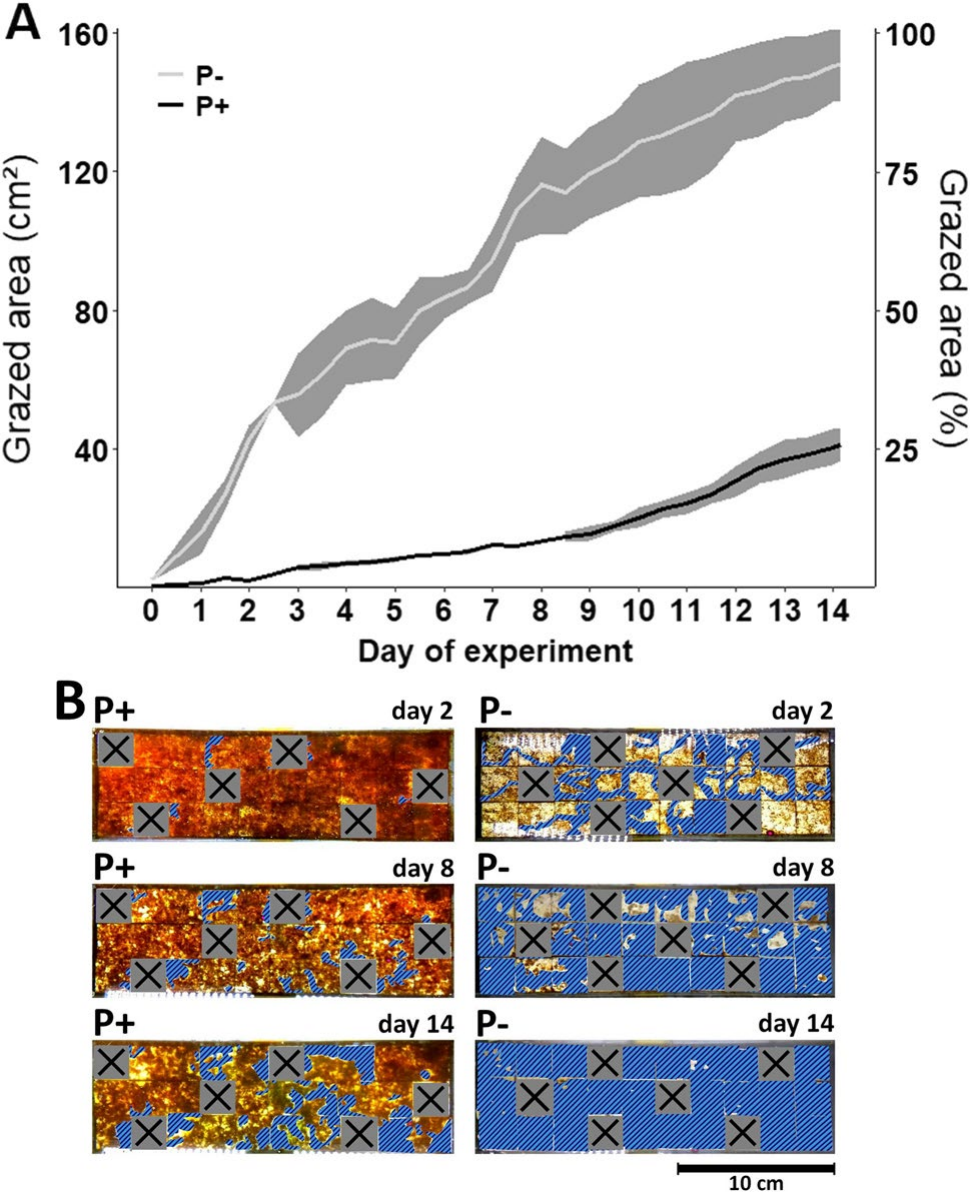
**A** Development of grazed area over time as absolute values and relative to total tile area, in the P+ (black line) and P− (white line) treatment in the presence of grazers. Values are mean ± SD (grey area) of *n* = 3 replicate flumes. At the end of the experiment (Day 14), grazed area was significantly larger in the P− than in the P+ treatment (student’s *t*-test; *t* = − 16.99, df = 4, *p* < 0.001). **B** Representative examples of ortho-photographs of periphyton cover from one P+ and P− flume, respectively, in the presence of grazers, at day 2, 8, and 14 of the experiment. Hatched areas represent grazed areas marked for quantification. Crossed out squares represent tiles that were removed in a randomised manner before test start for sampling and excluded from analysis

Phosphorus addition strongly altered the grazing range of *A. fluviatilis* and thus the periphyton area affected by grazing (Fig. 4). After 14 days of grazing, a periphyton cover of 151 ± 10 cm^−2^ (mean ± SD) was removed by *A. fluviatilis* in the P− treatment, whereas a periphyton cover of 41 ± 5 cm^−2^ was removed through grazing in the P + treatment, significantly less than in the P− treatment (student’s *t*-test; *t* = − 16.99, df = 4, *p* < 0.001; Fig. 4). In the P− treatment, 95 ± 6% of the periphyton cover was removed through grazing, as opposed to 26 ± 3% in the P+ treatment (Fig. 4).

### Feeding activity of *A. fluviatilis*

**Fig. 5:**
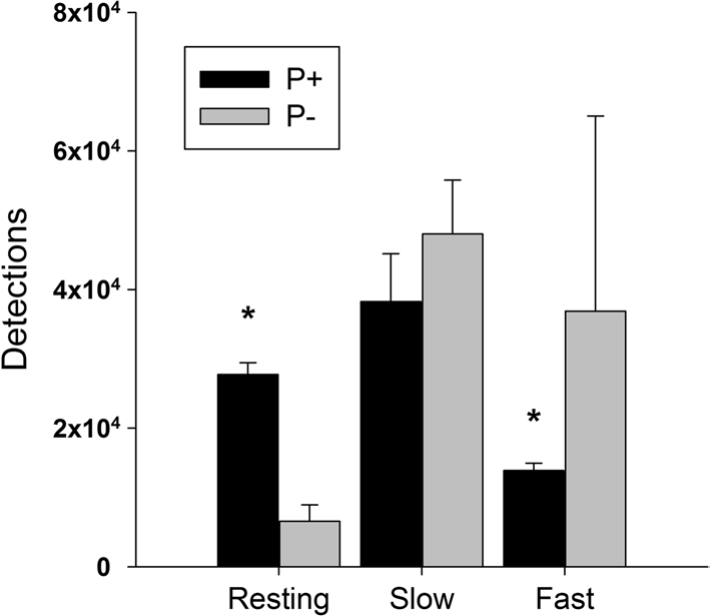
Video tracking detections of moving speeds performed by *A. fluviatilis* categorised as resting (0 to  < 0.4 cm h^−1^), slow (0.4–4 cm h^−1^), and fast (> 4 cm h^−1^) movements. Values are mean ± SD of *n* = 3 replicate flumes, in the P+ and P− treatment. *A. fluviatilis* was detected resting significantly more often in the P+ treatment compared to the P− treatment (student’s *t*-test; *t* = 12.5, df = 4, *p* < 0.001). Fast movements were detected significantly more often in the P− treatment than in the P+ treatment (Wilcoxon–Mann–Whitney test; *W* = 0, *p* = 0.04). There was no significant difference of slow movement detections between the two treatments (Wilcoxon–Mann–Whitney test; *W* = 2, *p* = 0.38). Asterisks indicate significant differences between the P+ and P− treatment

Under P+ conditions, *A. fluviatilis* rested significantly more often (27,741 ± 1718 detections, mean ± SD) compared to individuals kept under P- conditions (6550 ± 2380 detections) (student’s *t*-test; *t* = 12.50, df = 4, *p* < 0.001; Fig. 5). Movements classified as “slow” did not differ between treatments (Wilcoxon–Mann–Whitney test; *W* = 2, *p* = 0.38; Fig. 5). However, fast movements were detected significantly more often in the P− treatment (36,877 ± 28,190 detections) than in the P+ treatment (13,864 ± 1078 detections) (Wilcoxon–Mann–Whitney test; *W* = 0, *p* = 0.04; Fig. 5).

**Fig. 6:**
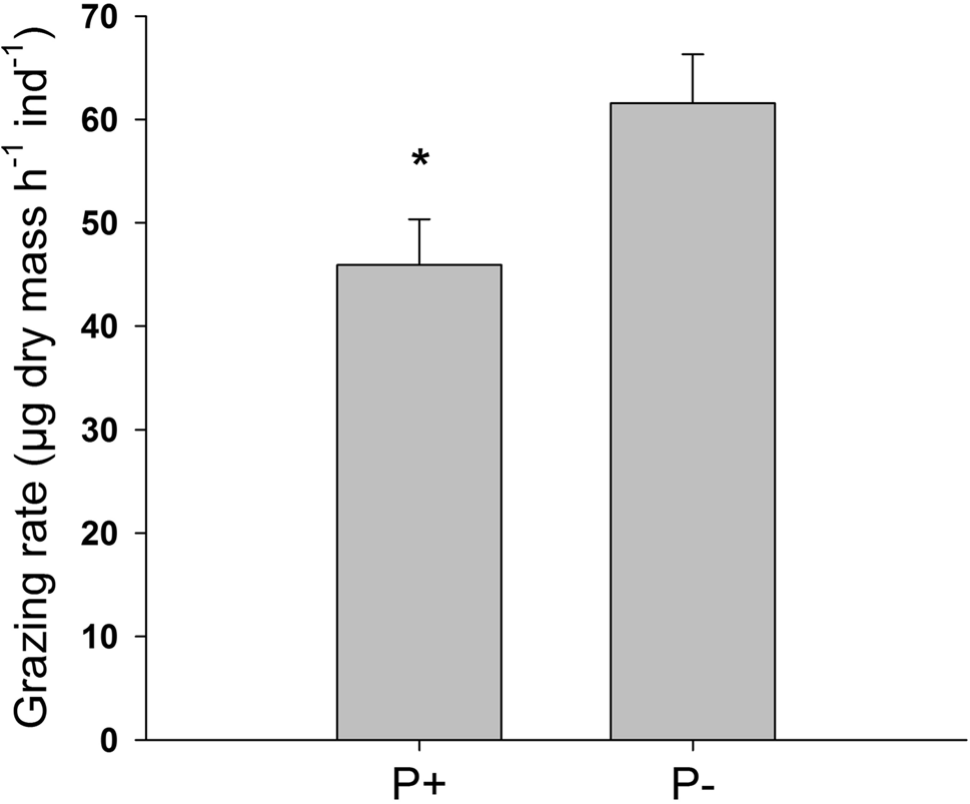
Grazing rates of *A. fluviatilis* of P+ and P− periphyton. Values are mean ± SD of *n* = 3 replicate flumes. Grazing rates in the P+ treatment were significantly lower compared to the P− treatment (student’s *t*-test; *t* = -4.19, df = 4, *p* = 0.02), indicated by an asterisk

*Ancylus fluviatilis* removed significantly more periphyton biomass in the P− compared to the P+ treatment (student’s *t*-test; *t* = − 4.19, df = 4, *p* = 0.02; Fig. 6). Average (± SD) grazing rates were 61.6 ± 4.73 µg h^−1^ ind^−1^ in the P− treatment, and 45.9 ± 4.42 µg h^−1^ ind^−1^ in the P+ treatment.

## Discussion

In this study, we combined a two-level factorial grazing experiment and the analysis of periphyton quantity and quality with video-based quantification of grazer behaviour. We present a three-step line of evidence, showing that bottom-up control of periphyton nutritional quality feeds back on the quantity and spatial arrangement of the algal community, mediated by changes in top-down control. First, phosphorus addition significantly increased algal biomass and the relative phosphorus content of periphyton prior to the introduction of grazers, thereby confirming hypothesis 1. This outcome is commonly observed with nutrient enrichment (e.g. Fanta et al. 2010; Hill et al. 2011; Wurtsbaugh et al. 2019). Second, the higher periphyton quantity and quality in the phosphorus-enriched flumes subsequently caused grazers to increase their resting times, whereas grazers were prompted to move at higher speed and cover wider areas in the P− flumes, most likely in search for food. Phosphorus addition therefore significantly decreased the number and speed of *A. fluviatilis*’ foraging movements, supporting our second hypothesis. Furthermore, food consumption rates were significantly higher in the P− flumes, suggesting that grazers may have increased consumption of nutrient-poor food to maximise nutrient intake. This behavioural strategy, known as compensatory feeding, has been previously observed in *A. fluviatilis* (Iannino et al. 2019) as well as in other aquatic (Stachowicz and Hay 1999; Cruz-Rivera and Hay 2000; Fink and von Elert 2006) and terrestrial herbivores (Lavoie and Oberhauser 2004; Berner et al. 2005). Finally, the greater movements and consumption rates in the P− flumes resulted in a significantly stronger removal of periphyton by grazing compared to the P+ flumes, supporting hypothesis 3. At the end of the experiment, the P- flumes had been grazed virtually everywhere, while the phosphorus-enriched flumes were still substantially covered with periphyton even in the presence of grazers.

Interestingly, the foraging activity of *A. fluviatilis* increased significantly in the P− even though the absolute nutrient depletion was moderate. The average algal C:P ratio in the P− treatment was slightly above 300, which is in the lower range of C:P values generally observed in oligotrophic environments (Stelzer and Lamberti 2001; Bowman et al. 2005; Fink et al. 2006). This relatively low C:P ratio, despite the very low phosphorus supply, may be explained by the moderate light availability above the flumes, which simulates the dense canopy cover in the adjacent section of the Holtemme River. The C:P values measured in the P− treatment were within range of C:P ratios measured in this stretch of the Holtemme (266-460, Weitere et al. 2021). Moreover, in the P− treatment, periphyton was composed of > 90% diatoms, which tend to maintain low C:P ratios compared to green algae and cyanobacteria (Quigg et al. 2003; Iannino et al. 2020). In the phosphorus-enriched treatment, nutrient addition led to a significant increase in the proportion of green algae, an effect frequently observed with phosphorus enrichment (Leland and Porter 2000; Whorley and Wehr 2016; Iannino et al. 2020). On the other hand, we observed no effects of grazing on periphyton C:P ratio and taxonomic composition, which appeared to be strictly bottom-up controlled in all treatments.

Our study provides evidence that the foraging movements of a stream benthic herbivore in relation to food quantity and quality are analogous to those of terrestrial ungulate herbivores such as bison and moose, whose residence times in food patches often increase with food availability and nutritional quality (Searle et al. 2005; Courant and Fortin 2012). In addition, similarly to *A. fluviatilis*, several herbivorous ungulate species have been observed to limit their foraging ranges when food sources are abundant (Owen-Smith et al. 2010). In turn, we here demonstrate that such foraging behaviour may eventually result in a feedback loop between reduced grazing activity and increased primary producer biomass under eutrophied conditions. Slower foraging movements in nutrient-enriched ecosystems may cause an excessive build-up of primary producer biomass in undisturbed areas, further constraining the spatial foraging ranges of herbivores that typically prefer to feed on young, thin vegetation. As a result, a more relaxed grazing pressure may eventually exacerbate the harmful consequences of eutrophication for ecosystem structure and functioning (Clark et al. 2017; Wurtsbaugh et al. 2019).

While a reduced top-down pressure is a short-term response to increased food quantity and quality, as shown here, an increase in resource availability is usually accompanied by an increase in consumer biomass in the long run. Moreover, nutrient deficiency in nutrient-poor environments may constrain herbivore growth and reproduction (Elser et al. 2000; Fink and von Elert 2006), thereby resulting in a weaker top-down control. However, nutrient enrichment often leads to reduced plant and algal diversity (Worm et al. 2002; Hautier et al. 2009; Groendahl and Fink 2017) and to dominance of less nutritious algal taxa in aquatic environments (Smith 2003; Iannino et al. 2020), which can also be harmful for herbivore growth and fitness (Müller-Navarra et al. 2000; Unsicker et al. 2010; Aquilino et al. 2012). Therefore, the reduced foraging movements of herbivores caused by eutrophication may be further modified by complex long-term outcomes that require further investigation.

Our findings may additionally give new insights into optimal foraging models, which predict that grazers should abandon a food patch when the rate of food and nutrient intake drops below the average intake of the whole environment (Bailey and Provenza 2008). According to the theorem, patch residence time will decrease as the overall habitat food quality increases (Pyke 2019), which may occur due to nutrient enrichment. However, as demonstrated in the present study, such an outcome may still result in more frequent movements in a nutrient-poor environment, where food resources are scarcer and therefore patches will be depleted faster. Furthermore, herbivores are often not fully aware of the overall resource availability of their surroundings. In an environment with abundant and nutrient-rich food sources, herbivores may decrease their foraging movements to save on energetic costs of locomotion and thus improve net energy intake, thereby affecting the strength of top-down control of primary producer biomass.

Bottom-up and top-down effects significantly interact with each other in the control of algal and plant communities (Proulx and Masumder 1998; Groendahl and Fink 2017; Iannino et al. 2019). As food quantity and quality are critical in mediating the feeding behaviour and distribution of herbivores, nutrient enrichment will have a profound impact on herbivore foraging movements, both in aquatic and terrestrial ecosystems. It has further been shown that such responses to poor quality can scale up through food webs (e.g. Jochum et al. 2017). The present study demonstrates that the effects of nutrient enrichment on primary producer biomass can be magnified by the reduced foraging movements of herbivores resulting from increased food availability and nutritional quality. As nutrient enrichment dramatically increases the productivity and nutrient content of plants and algae, elevated nutrient inputs may lead to a significantly greater primary producer biomass not only directly, but also indirectly by restricting herbivore movements to smaller spatial ranges. Eventually, the resulting feedback loop between weaker top-down control and increased primary production will significantly alter the spatial structure of plant and algal communities.

## Data Availability

The datasets used during the study are available from the corresponding author on request.

## Code availability

The code used for the study is available from the corresponding author on request.
